# Computed Tomographic Appearance of Massive Intestinal Ascariasis in a Pediatric Patient

**DOI:** 10.7759/cureus.116778

**Published:** 2026-09-23

**Authors:** Helen Rynor, Sai R Vulasala, Kevin Pierre, Ruben G Ortiz Cordero, Anthony I Zarka

**Affiliations:** 1 Radiology, University of Florida College of Medicine - Jacksonville, Jacksonville, USA; 2 Radiology, University of Florida College of Medicine, Gainesville, USA; 3 Pediatrics, Mayo Clinic Alix School of Medicine, Rochester, USA; 4 Pediatric Radiology, Nemours Children's Health System, Jacksonville, USA

**Keywords:** ascaris lumbricoides, computed tomographic, nematodes, parasitic roundworms, pediatric radiology

## Abstract

Ascaris lumbricoides is among the most common intestinal helminths worldwide but is encountered less frequently in the United States. Although uncomplicated ascariasis is diagnosed clinically and by stool examination, imaging becomes important when intestinal obstruction or another complication is suspected. We report a nine-year-old boy who had recently emigrated from Guatemala and was transferred from an outside emergency department for severe abdominal pain and concern for small bowel obstruction. Computed tomography (CT) demonstrated an extensive burden of elongated, curvilinear intraluminal structures throughout the small bowel, several containing central gas, with associated bowel dilatation. Notably, the initial outside interpretation favored fecalized bowel contents, and the diagnosis was recognized only after the history of vomited worms prompted a radiology addendum. Stool examination confirmed A. lumbricoides ova. The child was managed conservatively with albendazole and bowel cleanout, and serial abdominal radiographs demonstrated progressive reduction in the visible worm burden without surgery. This case illustrates both the striking imaging appearance of massive intestinal ascariasis and a clinically important diagnostic pitfall in its recognition.

## Introduction

Ascaris lumbricoides is one of the most prevalent human intestinal helminths, infecting hundreds of millions of people worldwide [1,2]. Infection predominates in tropical and subtropical regions and is acquired by ingestion of infective eggs from fecally contaminated soil. Following ingestion, larvae hatch in the intestine, migrate through the portal circulation and lungs, ascend the respiratory tract, are swallowed, and mature into adult worms in the small intestine [1,2]. Most infections are asymptomatic or cause nonspecific gastrointestinal symptoms, but high worm burdens occur disproportionately in children and may cause intestinal obstruction and, less commonly, volvulus, intussusception, or perforation. Migration into the biliary or pancreatic ducts may produce additional complications [3-5].

Imaging is not routinely required to diagnose uncomplicated ascariasis but is valuable when obstruction or another abdominal complication is suspected. We present the CT and serial radiographic findings of massive intestinal ascariasis in a nine-year-old boy evaluated for possible small bowel obstruction, emphasizing a diagnostic pitfall in which a heavy worm burden was initially mistaken for fecalized bowel contents.

## Case presentation

A nine-year-old boy who had recently emigrated from Guatemala was transferred from an outside emergency department for worsening abdominal pain and concern for possible small bowel obstruction. His mother reported two episodes of passage of a single worm in the stool over the preceding two months. Five days before admission, he developed severe abdominal pain, diarrhea, abdominal distention, and nonbloody, nonbilious emesis and subsequently became unable to tolerate food or fluids. The day before transfer, he vomited five worms, followed by one additional worm on the morning of admission.

CT of the abdomen and pelvis obtained at the referring emergency department for worsening abdominal pain was initially interpreted as demonstrating fecalized small bowel contents with possible small bowel obstruction, although no discrete transition point was identified. An addendum was subsequently issued after the interpreting radiologist was informed that the patient was vomiting worms. The radiologist then raised concern that the extensive curvilinear intraluminal structures represented worms. 

On arrival at our institution, the patient was afebrile and hemodynamically stable. Surgical examination demonstrated mild abdominal distention and mild lower abdominal tenderness, greater on the left, with voluntary guarding but no rebound tenderness or other peritoneal signs. Laboratory evaluation showed a white blood cell count of 8.58 K/µL without eosinophilia and a hemoglobin level of 11.6 g/dL. Serum albumin was 3.8 g/dL, and electrolytes, renal function, and liver tests were within normal limits.

On our review of the CT, numerous elongated and curvilinear intraluminal structures were present throughout the small bowel, several containing central foci of gas that further delineated their tubular morphology (Figure 1). Associated segmental bowel dilatation was present, but there was no discrete transition point and no evidence of intussusception, volvulus, ischemia, or perforation. The liver and gallbladder were normal, without biliary ductal dilatation or other evidence of hepatobiliary involvement. A small volume of free pelvic fluid was present.

**Figure 1:**
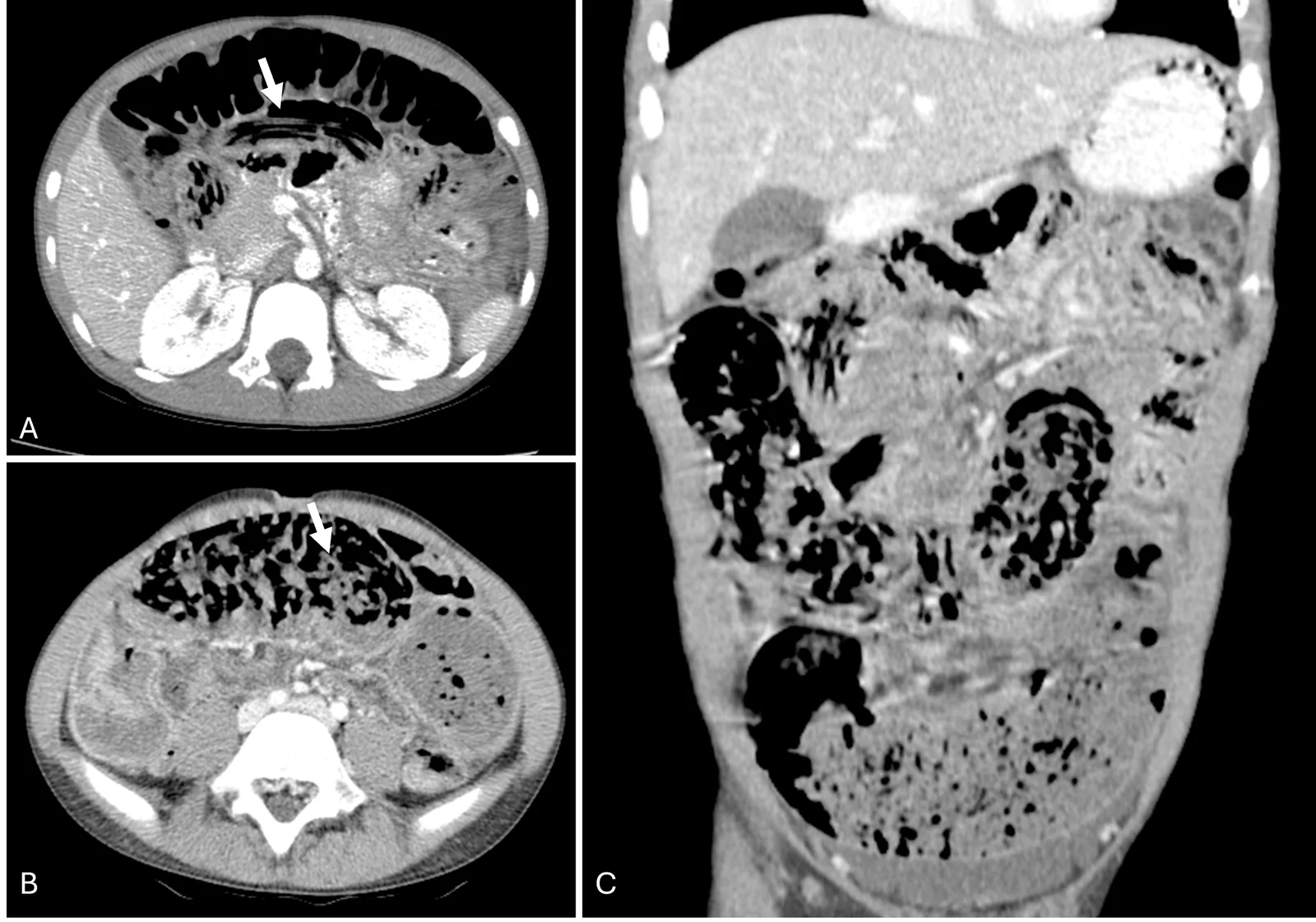
Contrast-enhanced CT demonstrates massive intestinal ascariasis. (A-B) Axial images through the abdomen show numerous elongated, curvilinear intraluminal structures filling multiple small-bowel loops, several containing a central longitudinal gas/contrast stripe corresponding to the worm's alimentary canal and accentuating their tubular morphology (arrows). (C) Coronal reformatted image demonstrates the extensive small-bowel worm burden with associated segmental bowel dilatation.

The patient was admitted, and infectious disease, gastroenterology, and surgery were consulted. Because he was hemodynamically stable without peritoneal signs or imaging evidence of a complication requiring intervention, conservative management was pursued. He received a single 400-mg enteral dose of albendazole. Stool examination confirmed A. lumbricoides ova.

His abdominal pain improved, and serial abdominal radiographs demonstrated progressive reduction in the visible curvilinear intraluminal densities (Figure 2). Gastroenterology and surgery determined that no procedural or surgical intervention was required, and bowel cleanout with polyethylene glycol was performed. At discharge, he was hemodynamically stable with good oral intake and output. The family was provided with a second 400-mg albendazole dose to administer approximately four weeks later if he had persistent gastrointestinal symptoms or continued to pass worms. No routine follow-up stool ova and parasites (O&P) testing to confirm eradication was documented; follow-up management was based on clinical symptoms and continued passage of worms.

**Figure 2:**
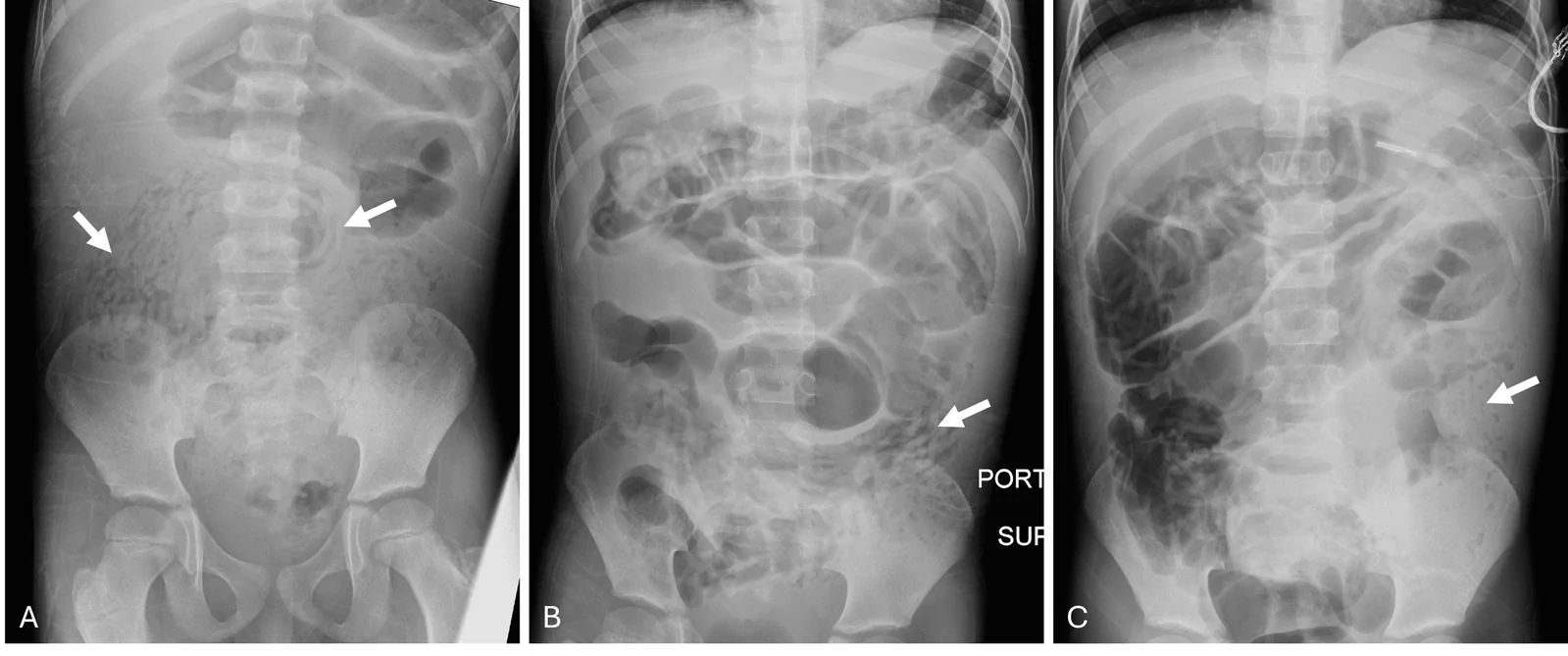
Serial supine abdominal radiographs of a child with heavy intestinal ascariasis, obtained on three consecutive days after initiation of anthelmintic therapy, demonstrating progressive treatment response. (A) Hospital day 2 (the day after albendazole was given): a confluent mass of tangled, curved soft-tissue strands is seen within distended small bowel loops (arrows), producing the characteristic "whirlpool" bolus-of-worms appearance of adult Ascaris lumbricoides [6]. (B) Hospital day 3: decrease in the intraluminal worm burden with residual curvilinear densities in the left lower quadrant (arrow), accompanied by increasing gaseous distention of the bowel. (C) Hospital day 4: further reduction in worm burden, though residual curvilinear densities persist in the left abdomen (arrow), with continued increase in luminal gas. The reciprocal relationship between decreasing worm burden and increasing luminal gas reflects re-aeration of loops previously packed with a worm bolus, paralleling clinical improvement.

## Discussion

Ascariasis remains a major global parasitic infection, particularly in tropical and subtropical regions. Most infections are asymptomatic, but heavy worm burdens occur disproportionately in children and can produce intestinal obstruction [1,5,6]. Importantly, this patient was not imaged simply to establish a diagnosis of uncomplicated ascariasis. CT had already been obtained at the referring emergency department for worsening abdominal pain, with possible bowel obstruction raised on the initial interpretation.

When imaging is required in children, ultrasonography is particularly useful because it avoids ionizing radiation and can demonstrate characteristic worm morphology, including elongated echogenic tubular structures with a central anechoic component, sometimes with visible movement on real-time imaging [4,6,7]. CT is more appropriately reserved for diagnostic uncertainty or suspected complications such as obstruction, ischemia, or perforation. On CT, worms may appear as elongated or curvilinear intraluminal structures [4,6,8]. In our patient, central gas within several worms further accentuated their tubular morphology.

Recent radiologic reviews have highlighted the multimodality appearance of ascariasis and other helminthic infections [6,8]. The extensive burden in our patient provides a particularly compelling CT demonstration of intestinal ascariasis, with numerous individually discernible tubular structures throughout the small bowel. The most instructive aspect of this case, however, was the diagnostic pitfall it produced. On the initial CT, the extensive intraluminal worm burden was interpreted as fecalized small bowel contents, with the diagnosis of ascariasis recognized only after the history of worm emesis was relayed to the interpreting radiologist. In regions where ascariasis is uncommon, radiologists may have limited firsthand experience with its imaging appearance, potentially contributing to diagnostic difficulty [8]. In our patient, the multiplicity and relatively uniform tubular morphology of the structures, with central gas in several worms accentuating their appearance, became particularly compelling once the relevant clinical history was known. The history of recent emigration from an endemic region and worms in both stool and emesis was decisive in reframing the imaging findings, underscoring the importance of timely communication of relevant clinical context.

Distinguishing a high worm burden with bowel dilatation from established mechanical obstruction is clinically important. Severe pediatric infection can result in a mass of worms causing intestinal obstruction, and complicated cases may require operative intervention [5,6]. In this patient, however, CT demonstrated no discrete transition point, volvulus, intussusception, ischemia, or perforation, and the surgical examination demonstrated no peritoneal signs. His symptoms improved with albendazole and bowel cleanout without operative intervention.

An additional noteworthy feature of this case is the radiographic documentation of treatment response. Serial abdominal radiographs demonstrated progressive reduction in the visible curvilinear intraluminal densities as the patient's symptoms improved. Although serial radiography is not routinely required to monitor uncomplicated ascariasis, in this case it provided a striking visual correlate of decreasing worm burden.

## Conclusions

Massive intestinal ascariasis can produce a distinctive imaging appearance and may present with bowel dilatation and clinical concern for obstruction. A heavy worm burden can mimic fecalized bowel contents, particularly when the relevant clinical history is not initially available. Imaging is not routinely required to diagnose uncomplicated ascariasis but is valuable for evaluating severe symptoms or suspected complications. In this case, CT demonstrated an extensive intraluminal worm burden, while serial abdominal radiographs provided a clear visual demonstration of decreasing worm burden following treatment. Recognition of the characteristic elongated, curvilinear intraluminal structures and integration of the clinical history can facilitate correct diagnosis and appropriate management.

## References

[1] (2026). About ascariasis. https://www.cdc.gov/sth/about/ascariasis.html.

[2] Jourdan PM, Lamberton PH, Fenwick A, Addiss DG (2018). Soil-transmitted helminth infections. Lancet.

[3] Ortega CD, Ogawa NY, Rocha MS, Blasbalg R, Caiado AH, Warmbrand G, Cerri GG (2010). Helminthic diseases in the abdomen: an epidemiologic and radiologic overview. Radiographics.

[4] Das CJ, Kumar J, Debnath J, Chaudhry A (2007). Imaging of ascariasis. Australas Radiol.

[5] Else KJ, Keiser J, Holland CV (2020). Whipworm and roundworm infections. Nat Rev Dis Primers.

[6] Cardoso MR, Sameshima YT, Del Campo Braojos Braga F, Gual F, Fernandes-Ferreira R, Souza AS (2025). Multimodality imaging of pediatric ascariasis. Pediatr Radiol.

[7] Chokkappan K, Subramanian M (2019). The inner tube sign in ascariasis. Abdom Radiol (NY).

[8] Solomon N, Calle F, Sorkun M (2025). Multimodality imaging of helminthic infections. Radiographics.

